# 

**Published:** 1828-10

**Authors:** 


22. *Necrology.* Died, in Paris within the present year, the celebrated Dr. Gall. He was a native of Germany, but had resided, latterly, in France. Whatever may be the fate of Dr. Gall's system of *Phrenology*, his researches into the anatomy of the brain, in conjunction with Dr. Spurzhiem, will entitle him to a high rank among those who have adventurously devoted themselves to inquiries into the structure of that most complicated organ. Previously to his death, Dr. G. was extensively employed in the practice of medicine in the French metropolis.

Died in Cincinnati, on the 14th inst. of Hydrothorax, Dr. E. H. Pierson, aged 57 years. Dr. P. was a native of Jersey, and had resided for the last 10 years in this city, where he was engaged in an extensive practice up to the week of his death. At the time of his decease, he was a Trustee of the Medical College of Ohio, and President of the Board of Health.

